# Retraction Note: TET2 inhibits tumorigenesis of breast cancer cells by regulating caspase-4

**DOI:** 10.1038/s41598-019-39690-5

**Published:** 2019-03-28

**Authors:** Xuguo Zhu, Shuangqi Li

**Affiliations:** Laboratory of Epigenetics, Institutes of Biomedical Sciences, Fudan University, Shanghai, 200032 China

Retraction of: *Scientific Reports* 10.1038/s41598-018-34462-z, published online 01 November 2018

This Article has been retracted because it was submitted by the corresponding author Zuguo Zhu without the knowledge or consent of the other listed author. Additionally, the majority of the individuals who directly contributed to the work were not included as authors.

Both listed authors agree to the retraction of the Article.

